# Supplementary material for: Identification of photoperiod-induced specific miRNAs in the adrenal glands of Sunite sheep (Ovis aries)
Source: Front Vet Sci. 2022 Jul 22;9:888207. doi: 10.3389/fvets.2022.888207 (PMC9354845; doi:10.3389/fvets.2022.888207)
Supplement: Supplementary file 1 [file Data_Sheet_1.ZIP › Supplementary file/miRDeep2 file.zip!/miRDeep2 file/Novel_Structure/LP42A2/Novel_621.pdf]

Provisional ID	:	NC_019471.2_24454
Score total	:	130.1
Score for star read(s)	:	3.9
Score for read counts	:	122
Score for mfe	:	2.6
Score for randfold	:	1.6
Score for cons. seed	:	
Total read count	:	251
Mature read count	:	239
Loop read count	:	0
Star read count	:	12

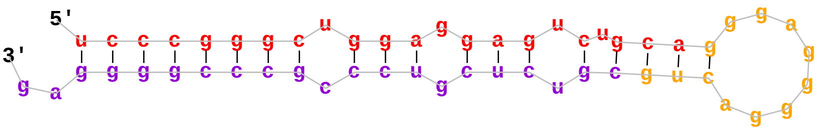

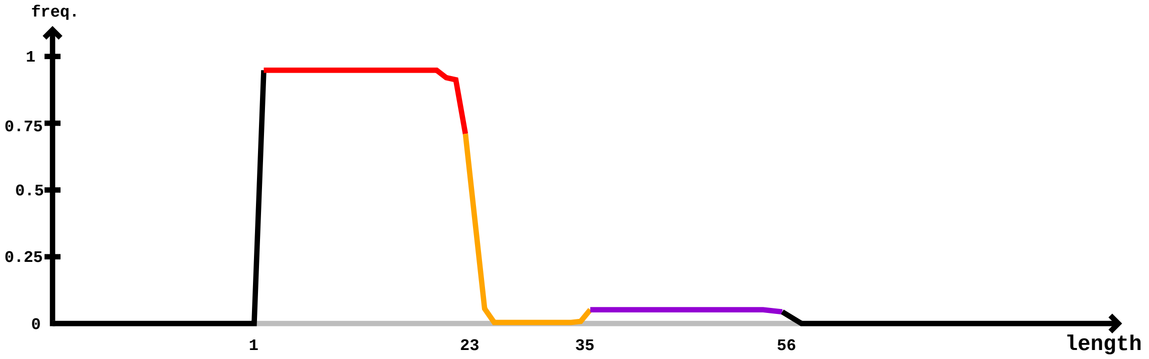

Mature Star

```
5'- ggaccugggcaggggcggggucccgggcuggaggagucugcagggaggggacugcgucucgucccgcccggggagaccgccccuugccgcucccaggacgguggauagc   -3'   obs
    ggaccugggcaggggcggggucccgggcuggaggagucugcagggaggggacugcgucucgucccgcccggggagaccgccccuugccgcucccaggacgguggauagc         exp
    .......(((((((((((..((((((((.(((.(((.(.((((........))))).))).))).))))))))...))))))).))))(((.(((......)))..)))   reads   mm   sample
    ....................ucccgggcuggaggagucu......................................................................   7       0    seq
    ....................ucccgggcuggaggagucug.....................................................................   2       0    seq
    ....................ucccgggcuggaggagucugc....................................................................   51      0    seq
    ....................ucccgggcuggaggagucugca...................................................................   118     0    seq
    ....................ucccgggcuggaggagucugcag..................................................................   48      0    seq
    ....................ucccgggcuggaggagucugcagg.................................................................   13      0    seq
    ...........................................ggaggggacugcgucucgucccgcccgggg....................................   1       0    seq
    .....................................................gcgucucgucccgcccggggaga.................................   1       0    seq
    ......................................................cgucucgucccgcccgggga...................................   1       0    seq
    ......................................................cgucucgucccgcccggggag..................................   7       0    seq
    ......................................................cgucucgucccgcccggggaga.................................   3       0    seq
```
